# Positive Youth Development and Wellbeing: Gender Differences

**DOI:** 10.3389/fpsyg.2021.641647

**Published:** 2021-07-20

**Authors:** Gina Tomé, Margarida Gaspar de Matos, Marta Reis, Diego Gomez-Baya, Filipa Coelhoso, Nora Wiium

**Affiliations:** ^1^Faculdade de Motricidade Humana (Equipa Aventura Social), Universidade de Lisboa, Lisbon, Portugal; ^2^Faculdade de Medicina, Instituto de Saúde Ambiental, Universidade de Lisboa, Lisbon, Portugal; ^3^Bolseira Pós Doutoramento FCT SFRH/BPD/108637/2015, Lisbon, Portugal; ^4^Bolseira Pós Doutoramento FCT SFRH/BPD/110905/2015, Lisbon, Portugal; ^5^Department of Social, Developmental and Educational Psychology, Universidad de Huelva, Huelva, Spain; ^6^ISCE - Instituto Superior de Lisboa e Vale do Tejo, Ramada, Portugal; ^7^Faculty of Psychology, University of Bergen, Bergen, Norway

**Keywords:** positive youth development, adolescents, well-being, gender differences, mental health

## Abstract

The five C’s of positive youth development (PYD) (competence, confidence, character, caring, and connection) have been associated with adaptive development among young people. Gender differences in young people’s wellbeing and mental health have been studied and analyzed, but the investigation into their association with the five C’s is still in its infancy. In the present study, we analyzed the influence of the five C’s on the wellbeing, more specifically, anxiety, social alienation, general wellbeing, physical symptoms, and psychological symptoms, of Portuguese adolescents, by gender. Participants were 5^th^–12th grade students attending public schools in Lisbon, Portugal. The questionnaire was administered to 384 adolescents. The results indicated important gender differences in young people’s wellbeing. The results revealed some differences between genders that should be considered in interventions that aim to promote the wellbeing of adolescents. On the other hand, confidence was positively associated with mental health and wellbeing for both boys and girls, skill should be promoted among young people, because it was competence that revealed the greatest association with wellbeing, among the variables analyzed.

## Introduction

Youth psychosocial development is influenced by individual and environmental/contextual factors associated with adolescent wellbeing. Several studies reported gender and age differences in adolescent wellbeing. Some analyses showed that, compared with boys, girls (especially older adolescent girls) increasingly report more emotional problems, internalizing problems, lower life satisfaction, and more frequent multiple health complaints (Gaspar et al., 2018; Cosma et al., 2020). A positive youth development (PYD) perspective focused on recognizing psychosocial strengths and providing social environments that contribute to the adolescent’s global development, including promoting wellbeing (Richardson et al., 2017). So, it seems important to understand how the adolescent’s positive development differentiates the impact on gender wellbeing, trying to promote the overall adolescent wellbeing.

Some authors have argued that the youth developmental transition to adulthood now has been extended to 29 years, lasting longer than ever (Arnett et al., 2014; Sawyer et al., 2018). Although developmental psychology has paid more attention to risk behaviors and emotional problems in this transition, it may be further characterized as a process of growth and building competence (Larson, 2008). Thus, research on youth development has followed a deficit perspective, which in turn has a guide policy design (Geldhof et al., 2014). In the last decade, the study of positive indicators has increased, since the results of intervention programs have underlined that promoting healthy youth development requires a strength-based approach, not only addressing risks and vulnerabilities (Kia-Keating et al., 2010).

Within the PYD framework, positive development can be operationalized by the “five Cs” of PYD—competence, confidence, connection, character, and caring (Bowers et al., 2010; Geldhof et al., 2014; Lerner et al., 2014). Competence represents an ability to navigate youth contexts effectively in order to achieve desired goals. Confidence arises when the navigation of one’s context results in feelings of personal agency and self-worth. Character represents the internalization of moral standards through repeated person-context relations as well as the behavioral manifestations of that moral code. Caring indicates developmentally and contextually appropriate levels of concern for others, and connection requires that the individual be embedded in, and supported by, a reliable and diverse social network (Geldhof et al., 2019).

These PYD domains are interconnected and young people require a healthy development in all of them to experience adaptive development (Geldhof et al., 2019).

Holsen et al. (2017) observed in a study involving 1,195 upper secondary school students (ages 16–19) in Norway and 839 participants of the 4-H Study of PYD in the United States, that the five C’s tended to correlate positively with indicators of adaptive development and negatively with maladaptive outcomes. The only unexpected finding was a positive correlation between caring and anxiety and depressive symptoms (Holsen et al., 2017).

Årdal et al. (2018) found that only the competence, confidence, and connection factors predicted school satisfaction. Competence, confidence, and connection fully mediated the effect of school empowerment on school satisfaction. In terms of gender, the five C’s were more strongly related to school empowerment and school satisfaction for girls than for boys (Årdal et al., 2018).

In Portugal, from 2014 the Social Adventure team was a pioneer in the implementation of a nationwide project called Dream Teens aiming at enhancing young people’s participation and active citizenship in the Portuguese context. The Dream Teens project used an innovative PYD approach that engaged Portuguese youth (aged 11–18 years). Participants from all over the country were empowered (1) to design and conduct research activities about their behaviors and about their life contexts and (2) to create ways to improve youth civic participation in their communities, while developing supportive interactions with adults and peers (Matos et al., 2015; Branquinho and Matos, 2018). A few other projects were derived from this, either developing youth positive development and participation throughout their empowerment (check all projects: methods, participants, and results at^1^).

Still in Portugal, Matos et al. (2017, 2018) performed two studies that analyzed the association between the five C’s of PYD and several psychosocial variables (i.e., resilience, self-regulation, anxiety, perception of academic abilities, goals, and expectations) in a sample of 2,700 young people with a mean age of 21 years and found a tendency for psychosocial variables to have a significant impact on total PYD and its five dimensions (competence, caring, character, confidence, and connection). And yet, Tomé et al. (2020) observed in their study that the five C’s were generally related to adolescents’ mental health and wellbeing. Specifically, confidence, connection, and competence were relevant features regarding young people’s wellbeing. That is, adolescents with higher levels of confidence, connection, and competence, had better wellbeing. So, ensuring that young people are thriving also means enhancing their mental health and wellbeing.

Recent research has consistently noted gender differences in psychological adjustment, lifestyles, and academic adjustment among young people (Gomez-Baya et al., 2019). In turn, Shakya et al. (2019) argued that sex-specific differences in health outcomes are caused by biological factors, or are socially driven through gender norms.

Ando et al. (2018) suggested that gender norms may influence gender differences in relation to issues related to mental health. The authors observed that gender norms inhibited intention of seeking help for depression more strongly in boys than in girls. Thus, they argued that actions should be considered against gender norms that presume that boys should solve their own problems (Ando et al., 2018). Kågesten et al. (2016) in their study verified that adolescents in different cultural settings commonly endorse norms that perpetuate gender inequalities, and that parents and peers are especially central in shaping such attitudes.

Furthermore, literature has consistently showed some gender differences in psychological adjustment and lifestyles during the youth life stage. Thus, more depression and anxiety have been observed in female youth and adolescents (Essau et al., 2010; McLean et al., 2011). However, more evidence is needed concerning gender differences in the indicators of positive adjustment, such as PYD. In the United States, Zimmerman et al. (2008) showed that female youth reported greater overall PYD than male youth. In Spain, some gender differences in PYD were also detected, such that male students presented higher scores in confidence and competence, and female students showed more connection, caring, and character (Gomez-Baya et al., 2021). These gender differences in PYD may partly explain differences in thriving indicators, for example, social contribution, school engagement, lifestyle, and mental health (Gomez-Baya et al., 2019). Thus, the examination of the associations between PYD and mental health in youth may require the analysis of gender differences and the exploration of the mechanisms implicated in each gender.

Regarding gender differences in the five C’s, Conway et al. (2015) found that girls scored higher than boys on caring, character, and connection, while boys scored higher on confidence and competence. Furthermore, Gomez-Baya et al. (2019) found that girls showed more connection, caring, and character, as well as more social engagement (i.e., more frequent help to friends and family, and more mentoring of others) and academic adjustment (i.e., more perceived performance and less boredom) than boys. Furthermore, boys showed greater scores in competence and confidence, less depressive and anxious symptoms, and more frequent physical activity than girls.

Considering the presented studies, it seems relevant to understand if there are gender differences for the five C’s impact on adolescent wellbeing. It is not intended to promote gender inequality, on the contrary, it is intended to promote the adolescent’s global positive development using effective strategies. So, the study aimed was to analyze the influence of the five C’s in wellbeing, more specifically, anxiety, social alienation, general wellbeing, physical symptoms, and psychological symptoms of Portuguese adolescents, by gender.

The hypotheses raised for this study are: There are gender differences for the analyzed variables; there are gender differences in the five C’s impact on adolescent wellbeing; girls scored higher than boys on caring, character, and connection; boys have better indicators of wellbeing than girls.

## Materials and Methods

### Procedure

Data for the present study are drawn from the ES’COOL Project, which arose from the gap in the promotion of mental health in a school context. The main goal of ES’COOL is to promote adolescents’ mental health through the capacity building of school teachers and school staff. The program aims at developing personal and social skills to prevent anxiety and depression symptoms and to promote resiliency, and self-regulation in adolescents. Training of teachers will allow not only an early detection of problems that affect adolescents’ mental health, but also an early intervention, which improves care effectiveness (Tomé et al., 2017, Tomé et al., 2018a, 2019).

To evaluate the teacher training impact, teachers were asked to send a link to the questionnaire to their students at the beginning and a month after the training was completed. Teachers were asked to choose students without taking into account any particular characteristics, only the Student’s motivation to participate in the study (convenience sample). The questionnaire link was sent only to students who agreed to participate. The inclusion criterion of the study was to be a student of one of the teachers who was conducting the training.

The Student’s questionnaire consisted of questions about their mental health, wellbeing, school and teacher relationships, and the PYD questionnaire. Both questionnaires (teachers and adolescents) were submitted and approved by the Santa Maria Hospital Ethics Committee, Portugal, and informed consent was obtained from the caregivers in the case of adolescents under the age of 18 years (Tomé et al., 2017, Tomé et al., 2018a, 2019).

### Participants

The sample comprised students from teachers who participated in the ES’COOL training project (escool.pt). The questionnaire was administered to 384 adolescents (total sample that completed the pre-test), 53.4% were boys, aged between 10 and 20 years (*M* = 15.3, *SD* = 2.3). Participants were 5th–12th grade students attending national public schools (see Table 1; Tomé et al., 2017, Tomé et al., 2018a, 2019).

**TABLE 1:** Participants’ description.

	*N*	%	*M*	*SD*	*Range*
**Sample**					
**Gender**					
Boys	205	53.4			
Girls	179	46.6			
**Age**	384	100	15.3	2.3	10–20
**Grade**					
5th grade	8	2.1			
6th grade	40	10.4			
7th grade	23	6			
8th grade	25	6.5			
9th grade	68	17.7			
10th grade	84	21.9			
11th grade	68	17.7			
12th grade	68	17.7			
**Nationality**					
Portuguese	368	95.8			
Other	16	4.2			

### Instrument

The original version of the PYD questionnaire was developed with data from the 4-H Study (Lerner et al., 2005; Jelicic et al., 2007; Bowers et al., 2010) with samples of adolescents aged 10–16 years.

A shorter version of the PYD short form (PYD-SF) was later constructed. The scale was reduced from 78 to 34 items and translated into Portuguese (Geldhof et al., 2014). In this study, the version with 34 items was used.

Wellbeing was measured by the Kidscreen scale, a 10-item scale with options: 1-Never; 2-Rarely; 3-Quite often; 4-Very often; 5-Always; the higher the score the higher the wellbeing (Gaspar et al., 2012), with an internal consistency index of 0.84 (Gaspar and Matos, 2010). Alienation was measured by the Alienation Scale, validated for the Portuguese population in a previous study of Tomé et al. (2018a). The scale assesses social alienation level, with 10 items, with answer options ranging from 1- it is always true to 5- it is never true. After Portuguese validation, the scale was composed of three subscales: instability (four items), isolation (two items), and demotivation (four items) and a full scale (10 items). The higher the score the higher the social alienation (Tomé et al., 2018b).

Anxiety was measured by The Multidimensional Anxiety Scale for Children (MASC); validated for the Portuguese population in a previous study of Salvador and colleagues (Salvador et al., 2015; Tomé et al., 2020). This scale consists of 39 questions, with answer options ranging between 0- never or almost never true to 3- it is often true. After Portuguese validation, the scale was composed of five subscales: somatic anxiety (12 items), danger avoidance (9 items), separation anxiety (9 items), and social anxiety (9 items) and a full scale (39 items). The higher the score the higher the anxiety (Salvador et al., 2015; Tomé et al., 2020).

Psychological symptoms were assessed with the questions used in the HBSC study protocol for health symptoms; the higher the score the higher the symptoms (sadness, irritation or bad temper, nervousness, tiredness and exhaustion, difficulties in getting to sleep, and self-harm) (Matos et al., 2015).

The selected variables to assess the adolescents’ wellbeing were used in previous studies and proved to be good indicators of wellbeing among adolescents (see Gaspar and Matos, 2010; Geldhof et al., 2014; Matos et al., 2015; Salvador et al., 2015; Tomé et al., 2018a, Tomé et al., 2020).

### Statistical Analysis

Data were analyzed through the statistics program SPSS 24. For data analysis, mean comparison (ANOVA) and multiple linear regressions were performed.

## Results

The internal consistency values of the subscales used in this study are good, with Cronbach’s alphas ranging from α = 0.73 to α = 0.88 and can be found in the previous article by Tomé et al. (2020).

Regarding gender differences in the five C’s observed in the ANOVA test, boys scored higher on competence (*M* = 22.5, *SD* = 3.9), confidence (*M* = 23.4, *SD* = 4), and connection (*M* = 31.2, *SD* = 5.1), when compared to girls. For the alienation scale, the differences were not significant for the total scale (alienation total) and isolation and demotivation subscales. For the instability subscale, girls scored higher (*M* = 10.7, *SD* = 2.6) when compared to boys. For the anxiety scale, the differences were not significant for the danger avoidance subscale and separation anxiety subscale, while for the anxiety total (*M* = 53.2, *SD* = 15.9), somatic anxiety (*M* = 15.7, *SD* = 3.9), and social anxiety (*M* = 13.9, *SD* = 6.5) girls had a higher average. For wellbeing (Kidscreen), boys (*M* = 38.2, *SD* = 7.2) scored higher when compared to girls. Finally, for physical symptoms (*M* = 10.5, *SD* = 4.5) and psychological symptoms (*M* = 12.7, *SD* = 5.5), girls had a higher average (see Table 2).

**TABLE 2:** Gender differences—five C‘s and wellbeing (ANOVAS).

PYD five C’s	Gender	Boys			Girls				
		*N*	*M*	*SD*	*N*	*M*	*SD*	*t*	*p*
	Competence	205	**22.5**	3.9	179	19.5	4.3	−7.219	**0.000**
	Confidence	205	**23.4**	4	179	20.8	5.6	−5.331	**0.000**
	Character	205	31	4.5	179	31.5	4.3	1.118	0.264
	Caring	205	23.9	4.8	179	24.6	4.6	1.280	0.201
	Connection	205	**31.2**	5.1	179	29.3	5.3	−3.607	**0.000**
**Alienation scale**	**Gender**	**Boys**			**Girls**				
		*N*	*M*	*SD*	*N*	*M*	*SD*	*t*	*p*
	Alienation (total)	205	27.5	4.9	179	28.4	6.2	1.474	0.141
	Instability	205	9.8	2.8	179	**10.7**	2.6	3.047	**0.002**
	Isolation	205	5.4	2.5	179	5.7	2.6	1.025	0.306
	Demotivation	205	12.3	4.7	179	12	3.8	−0.632	0.528
**Anxiety (MASC)**	**Gender**	**Boys**			**Girls**				
		*N*	*M*	*SD*	*N*	*M*	*SD*	*t*	*p*
	Anxiety (total)	205	48.3	21.4	179	**53.2**	15.9	2.562	**0.011**
	Somatic anxiety	205	12.1	7.9	179	**15.7**	3.9	2.983	**0.003**
	Danger avoidance	205	15.1	4.7	179	15.7	3.9	1.282	0.201
	Separation anxiety	205	8.6	5.7	179	9.2	4.3	1.142	0.254
	Social anxiety	205	12.4	6.4	179	**13.9**	6.5	2.315	**0.021**
**Gender**		**Boys**			**Girls**				
		*N*	*M*	*SD*	*N*	*M*	*SD*	*t*	*p*
Kidscreen (wellbeing)		198	**38.2**	6.5	178	35	7.2	−4.528	0.**000**
**Gender**		**Boys**			**Girls**				
		*N*	*M*	*SD*	*N*	*M*	*SD*	*t*	*p*
Physical symptoms		202	8.3	3.9	178	**10.5**	4.5	−6.983	0.**000**
Psychological symptoms		202	9.2	4.3	178	**12.7**	5.5	5.230	**0.000**

*Bold values correspond to the significant and highest values.*

To understand the predictive effect of the five C’S in wellbeing for boys and girls, multiple linear regression analyses were conducted separately for the two genders. In the models, only significant variables were used in the ANOVA analysis.

We chose to perform regression models separated by gender, as it was considered that it would demonstrate more clearly the real differences between genders, which would not be so clear if we included gender in the regression models. The inclusion of genders in the regression model would reveal the weight of gender in a multi-group model, which is not the intended objective.

In the boys’ model, the regression equation for instability (alienation subscale) explained 1% of the variance (*R*^2^ = 0.016). This model was not adjusted or significant. For anxiety (total), the variance explained only 5% (*R*^2^ = 0.050) of the general model, through competence (high competence score was associated with more probability of anxiety). For somatic anxiety, the variance explained was about 2% (*R*^2^ = 0.016) of the general model, through the following variables, competence (high competence, more probability of somatic anxiety) and confidence (high confidence, lesser probability of somatic anxiety). For social anxiety, the variance explained was 9% (*R*^2^ = 0.089), through the following variables, competence (high competence was associated with more probability of social anxiety), confidence (high confidence, lesser probability of social anxiety), and caring (high caring, higher probability of social anxiety). For wellbeing (Kidscreen), the variance explained was 47% (*R*^2^ = 0.470), through the variables confidence (high confidence, more probability of wellbeing), character (high character, more probability of wellbeing), and connection (high connection, higher probability of wellbeing). For physical symptoms, the variance explained was 6% (*R*^2^ = 0.058). This model was not adjusted or significant. Finally, for psychological symptoms, the variance explained was 18% (*R*^2^ = 0.181), through the variable confidence (less confidence, more probability of psychological symptoms) (see Table 3).

**TABLE 3:** Multiple linear regression—boys.

Instability (alienation)	Variable included	*β*	*t*	*p*	*R^2^_*a*_*	F(model fit)*
	Competence	0.028	0.252	0.801	0.016	0.363
	Confidence	–0.104	–0.907	0.366		
	Character	–0.010	–0.093	0.926		
	Caring	0.049	0.595	0.559		
	Connection	0.088	0.845	0.399		
Anxiety (total)	**Variable included**	***β***	***T***	***p***	***R^2^_*a*_***	F(model fit)*
	Competence	0.212	2.003	0.046	0.050	3.148
	Confidence	–0.213	–1.932	0.055		
	Character	0.062	0.622	0.535		
	Caring	0.144	1.767	0.079		
	Connection	0.062	0.614	0.540		
Somatic anxiety	**Variable included**	***β***	***t***	***p***	***R^2^_*a*_***	F(model fit)*
	Competence	0.278	2.586	0.010	0.016	1.753
	Confidence	–0.235	–2.092	0.038		
	Character	0.006	0.055	0.956		
	Caring	0.032	0.390	0.697		
	Connection	0.025	0.242	0.809		
Social anxiety	**Variable included**	***β***	***T***	***p***	***R^2^_*a*_***	F(model fit)*
	Competence	0.259	2.500	0.013	0.089	5.007
	Confidence	–0.411	–3.802	0.000		
	Character	0.054	0.550	0.583		
	Caring	0.206	2.571	0.011		
	Connection	0.022	0.227	0.821		
Kidscreen (wellbeing)	**Variable included**	***β***	***t***	***p***	***R^2^_*a*_***	F(model fit)*
	Competence	0.001	0.017	0.987	0.470	37.079
	Confidence	0.404	4.867	0.000		
	Character	0.167	2.211	0.028		
	Caring	0.007	0.122	0.903		
	Connection	0.224	2.964	0.003		
Physical symptoms	**Variable included**	***β***	***t***	***p***	***R^2^_*a*_***	F(model fit)*
	Competence	0.124	1.148	0.252	0.058	3.474
	Confidence	–0.167	–1.506	0.134		
	Character	–0.067	–0.868	0.386		
	Caring	–0.054	–0.666	0.506		
	Connection	–0.136	–1.351	0.178		
Psychological symptoms	**Variable included**	***β***	***t***	***p***	***R^2^_*a*_***	F(model fit)*
	Competence	0.174	1.735	0.084	0.181	9.893
	Confidence	–0.410	–3.954	0.000		
	Character	–0.182	–1.947	0.053		
	Caring	0.022	0.294	0.769		
	Connection	–0.054	–0.577	0.565		

*Statistically significant values.

In the girls’ model, the regression equation for instability (alienation subscale) explained 4% of the variance (*R*^2^ = 0.044) through the variable competence (less competence, more probability of instability). For anxiety (total), the variance explained was 8% (*R*^2^ = 0.083), through caring (high caring, more probability of anxiety). For somatic anxiety, variance explained was 12% (*R*^2^ = 0.116), through the variable connection (less connection, more probability of somatic anxiety). For social anxiety, the variance explained was 19% (*R*^2^ = 0.190), through the following variables, confidence (less confidence, more probability of social anxiety) and caring (high caring, higher probability of social anxiety). For wellbeing (Kidscreen), the variance explained was 60% (*R*^2^ = 0.600), through the following variables, competence (high competence, more probability of wellbeing), confidence (high confidence, more probability of wellbeing), and connection (high connection, higher probability of wellbeing). For physical symptoms the variance explained was 18% (*R*^2^ = 0.177) through the variables’ confidence (less confidence, more probability of physical symptoms) and connection (less connection, higher probability of physical symptoms). Finally, for psychological symptoms, the variance explained was 34% (*R*^2^ = 0.336), through the variables’ confidence (less confidence, more probability of psychological symptoms) and connection (less connection, more probability of psychological symptoms) (see Table 4).

**TABLE 4:** Multiple linear regression—girls.

Instability (alienation)	Variable included	*β*	*t*	*p*	*R^2^_*a*_*	F(model fit)*
	Competence	–0.243	–2.215	0.028	0.044	2.633
	Confidence	0.014	0.129	0.897		
	Character	0.075	0.757	0.450		
	Caring	0.036	0.400	0.669		
	Connection	–0.087	–0.855	0.3		
Anxiety (total)	**Variable included**	***β***	***T***	***p***	***R^2^_*a*_***	F(model fit)*
	Competence	–0.171	–1.589	0.114	0.083	4.204
	Confidence	–0.136	–1.309	0.192		
	Character	0.026	0.284	0.777		
	Caring	0.276	2.977	0.003		
	Connection	0.018	0.178	0.859		
Somatic anxiety	**Variable included**	***β***	***T***	***p***	***R^2^_*a*_***	F(model fit)*
	Competence	–0.146	–1.384	0.168	0.116	5.652
	Confidence	–0.139	–1.360	0.176		
	Character	0.085	0.889	0.375		
	Caring	0.122	1.341	0.162		
	Connection	–0.201	–2.054	0.041		
Social anxiety	**Variable included**	***β***	***t***	***p***	***R^2^_*a*_***	F(model fit)*
	Competence	–0.172	–1.698	0.091	0.190	9.357
	Confidence	–0.343	–3.502	0.001		
	Character	–0.122	–1.335	0.184		
	Caring	0.305	3.505	0.001		
	Connection	0.110	1.176	0.241		
Kidscreen (wellbeing)	**Variable included**	***β***	***T***	***p***	***R^2^_*a*_***	F(model fit)*
	Competence	0.198	2.779	0.006	0.600	54.043
	Confidence	0.332	4.806	0.000		
	Character	0.109	1.691	0.093		
	Caring	–0.087	–1.406	0.161		
	Connection	0.341	5.176	0.000		
Physical symptoms	**Variable included**	***β***	***t***	***p***	***R^2^_*a*_***	F(model fit)*
	Competence	0.028	0.271	0.787	0.177	8.626
	Confidence	–0.234	–2.364	0.019		
	Character	0.106	1.144	0.254		
	Caring	0.049	0.560	0.576		
	Connection	–0.346	–3.664	0.000		
Psychological symptoms	**Variable included**	***β***	***T***	***p***	***R^2^_*a*_***	F(model fit)*
	Competence	–0.003	–0.035	0.972	0.336	18.912
	Confidence	–0.360	–4.052	0.000		
	Character	–0.042	–0.507	0.613		
	Caring	0.096	1.211	0.227		
	Connection	–0.316	–3.722	0.000		

*Statistically significant values.

## Discussion

The five C’s have been observed to be associated with adaptive development among young people (Geldhof et al., 2019). Gender differences between young people’s wellbeing or mental health have been studied and analyzed, but research on their association with the five C’s is scarce. The aim of the present study was to analyze the influence of the five C’s on the wellbeing of Portuguese adolescents, by gender.

Considering the five C’s influence, the results indicated important gender differences for young people’s wellbeing. For boys, competence had a negative influence on anxiety (i.e., the higher the competence, the more anxiety symptoms boys had), while for girls this influence was positive, for example, for instability, high competence was associated with less instability, and for wellbeing, high competence was associated with enhanced wellbeing.

Character was associated with wellbeing for boys but not for girls, while caring emerged as a poor predictor of both boys’ and girls’ mental health. The current finding on caring is in line with the studies by Holsen et al. (2017) and Tomé et al. (2020) that identified that caring positively correlated with some malaise indicators. Caring indicated developmentally and contextually appropriate levels of concern for some youth (Geldhof et al., 2019), while others tend to develop inappropriate levels of concern, thus, promoting increased anxiety symptoms. This topic should be further investigated.

Connection appeared to be of greater importance for girls than boys. Whereas girls’ connection was associated with less somatic anxiety, fewer physical symptoms, fewer psychological symptoms, and high wellbeing, boys’ connection was only a predictor of high wellbeing. Shakya et al. (2019) argued that some sex-specific differences in health outcomes are caused by biological factors, while others are socially driven through gender norms. These results raise the question of whether social norms can have a strong impact on boys, inhibiting the positive influence of connection on their wellbeing and mental health. This topic should be addressed in research on mental health and wellbeing.

Apparently, confidence was the strongest predictor of mental health and wellbeing for both boys and girls, revealing an essential characteristic that should be promoted among young people.

Although it is also important in boys’ development, it was observed that the five C’s appear to have a greater influence on girls’ wellbeing and mental health. These results go in the same direction as those described by authors like Ando et al. (2018) and Shakya et al. (2019) who infer that social norms influence the differences between adolescents’ health behaviors. Thus, interventions must be developed in order to promote the wellbeing and mental health of young people, taking into account these gender differences.

In future studies it will be important to analyze whether these results have changed in the final questionnaire.

### Limitations

The main limitations of this study are related to the sample being a convenience sample and the study being a correlational type of study. Another limitation of the study is the lack of socio-demographic references of the sample which does not allow for the generalization of the results.

Main findings:

- The main differences found between the genders were: For boys, competence had a negative influence on anxiety, while for girls this influence was positive;
- Character was associated with wellbeing for boys but not for girls;
- Caring emerged as a poor predictor of both boys’ and girls’ mental health;
- Connection appeared to be of greater importance for girls than boys;
- Confidence appeared to have the greatest impact on boys’ and girls’ wellbeing and mental health.

## Data Availability Statement

The raw data supporting the conclusions of this article will be made available by the authors, without undue reservation.

## Ethics Statement

The studies involving human participants were reviewed and approved by the Santa Maria Hospital Ethics Committee, Portugal. Written informed consent to participate in the study was provided by the participants, and where necessary, the participants’ legal guardian/next of kin.

## Author Contributions

GT: data analysis. MG: work coordinator and manuscript reviewer. MR: support in literature review. DG-B: support in data analysis. FC: support in literature review. NW: support in manuscript review. All authors contributed to the article and approved the submitted version.

## Conflict of Interest

The authors declare that the research was conducted in the absence of any commercial or financial relationships that could be construed as a potential conflict of interest.

## Footnotes

1 www.aventurasocial.com
